# Vemurafenib Combined With Trametinib Significantly Benefits the Survival of a Patient With Stage IV Pancreatic Ductal Adenocarcinoma With BRAF V600E Mutation: A Case Report

**DOI:** 10.3389/fonc.2021.801320

**Published:** 2022-01-25

**Authors:** Ziyao Wang, Du He, Chen Chen, Xubao Liu, Nengwen Ke

**Affiliations:** ^1^ Department of Pancreatic Surgery, West China Hospital, Sichuan University, Chengdu, China; ^2^ Department of Pathology, West China Hospital, Sichuan University, Chengdu, China; ^3^ Department of Radiology, The First People’s Hospital of Chengdu, Chengdu, China

**Keywords:** vemurafenib, trametinib, BRAF V600E, PDAC, survival, benefit

## Abstract

Vemurafenib and trametinib have a lot of successful experiences in the treatment of unresectable or metastatic melanoma with BRAF V600E mutation. However, they have not been reported in the treatment of advanced pancreatic ductal adenocarcinoma (PDAC). We report here a 66-year-old male who was diagnosed as PDAC with multiple metastases of the abdominal cavity and liver according to pathological examination. After three cycles of gemcitabine plus nab-paclitaxel (GA) regimen chemotherapy, the liver metastasis of the patient progressed, and the patient could not continue to receive chemotherapy because of poor physical condition. BRAF V600E mutation was found by genetic detection in this patient, so targeted therapy with vemurafenib combined with trametinib was performed and the follow-up period was up to 24 months. To the best of our knowledge, this is a rare report that patients with stage IV PDAC with BRAF V600E mutation can receive significantly survival benefits from targeted therapy with vemurafenib combined with trametinib. This report provides experience for the use of these two drugs in patients with advanced PDAC.

## Introduction

The incidence of pancreatic ductal adenocarcinoma (PDAC) is increasing at a rate of 0.5%–1% per year. It is estimated that pancreatic cancer will become the second-leading cause of cancer death in the United States by 2030, and 50% of PDAC patients have already metastasized at diagnosis (1, 2). The current first-line chemotherapy treatment for metastatic patients is gemcitabine plus nab-paclitaxel (GA) or modified FOLFIRINOX regimen (1). GA regimen is a suitable choice for the elderly or poor physical condition patients (3), but its curative effect is not optimistic (4).

Patients with advanced PDAC are in urgent need of new treatment. Erlotinib, a tyrosine kinase inhibitor of epidermal growth factor receptor, is considered to be a promising drug for the treatment of advanced pancreatic cancer. Nevertheless, compared with gemcitabine alone, erlotinib combined with gemcitabine did not lead to a significant increase in progression-free survival (PFS) and overall survival (OS) (3, 5). Olaparib, a poly(adenosine diphosphate [ADB]-ribose) polymerase inhibitor, improved PFS but not OS in patients with BRCA1/2 mutation and metastatic PDAC after platinum-based chemotherapy (6). However, germline or somatic mutations of the BRCA1/2 exist only in 5% of patients with pancreatic cancer (7). Therefore, diverse treatments for advanced pancreatic cancer are needed.

Whole-exome sequencing showed that there were 92% PDAC patients who have *KRAS* mutation, and the mutation rate of BRAF V600E in KRAS wild-type patients was about 3%, which was mutually exclusive with KRAS mutations (8). This mutation greatly increased the activity of many carcinogenic-associated kinases and induced the proliferation of cancer cells (8). This mutation occurs in about half of melanomas and is prevalent in many other cancers (9). Vemurafenib affects the transduction of RAF signal pathway in BRAF mutant cells by affecting the dimerization of RAF, which plays an important role in the treatment of metastatic melanoma (10). However, since the drug abnormally activates the MAPK pathway in active cells driven by RAF (or other upstream signals), it is necessary to combine MEK inhibitors to overcome the drug resistance (11). Trametinib can non-competitively inhibit the activation and kinase activity of MEK1 and MEK2 (12). In combination with BRAF inhibitors, it delays drug resistance due to reactivation of the MAPK pathway during BRAF or MEK monotherapy and ultimately prolongs patient survival (11). To sum up, we report a case of vemurafenib combined with trametinib targeting therapy for advanced PDAC patients with BRAF V600E mutations after failed chemotherapy with the GA regimen. This patient benefited significantly from this combined targeting therapy.

## Case Description

**Figure 1:**
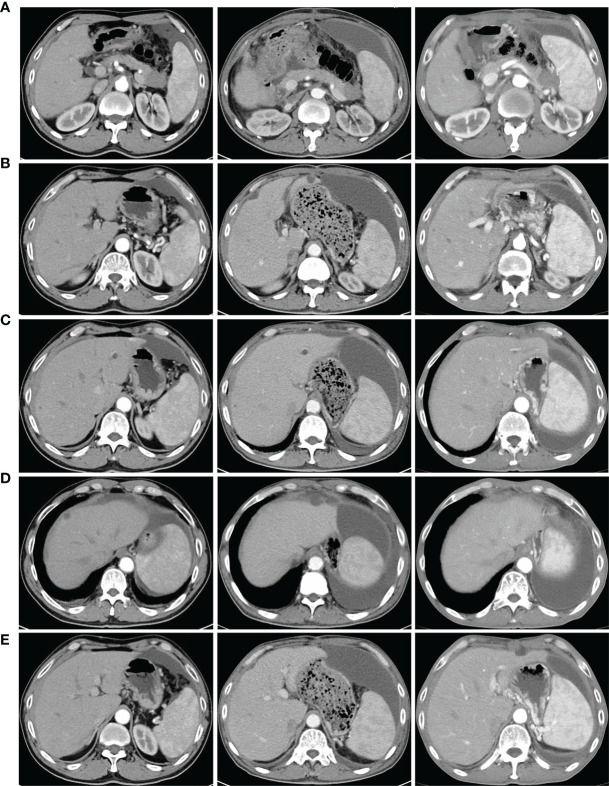
Contrast-enhanced CT of the whole abdomen and pelvic cavity of the patient in different periods. Enhanced scanning was performed using Omnipaque (300 mg/ml) with an injection rate of 3 ml/s. The arterial phase was scanned 28–30 s after injection, the portal phase 48–50 s, and the delayed phase 60–70 s. **(A)** Primary tumor: from left to right are preoperative, after chemotherapy, and after targeted treatment (the following sequence is from left to right). **(B)** No metastases before surgery, new metastases after chemotherapy, and metastases disappeared after target therapy. **(C)** Preoperative metastases, metastases increased after chemotherapy and decreased after targeted therapy. **(D)** Preoperative metastases, new metastases after chemotherapy and disappeared after targeted therapy. **(E)** No metastasis here before surgery, no metastasis here after chemotherapy, and new recurrence after targeted therapy.

**Figure 2:**
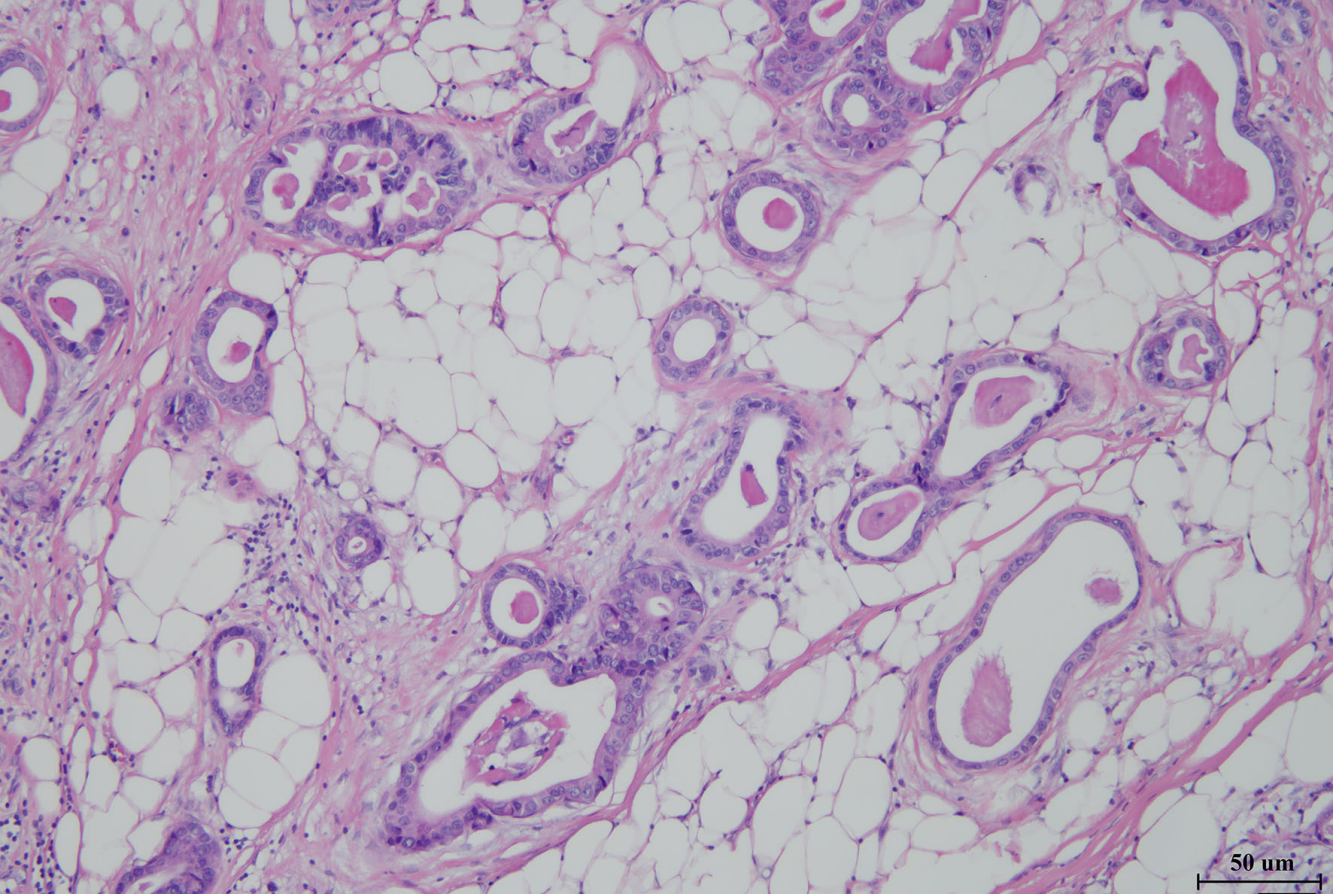
Hematoxylin–eosin staining image of metastases from the abdominal cavity (×100) of the patient, showing adenocarcinoma derived from pancreatic ducts.

**Figure 3:**
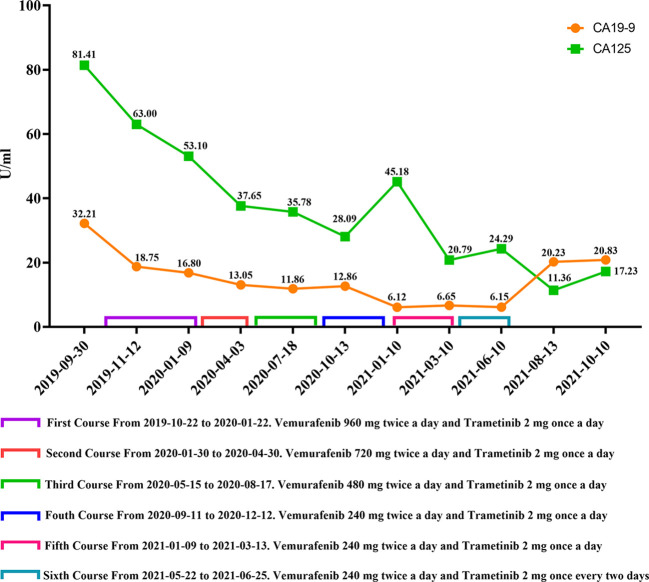
Changes in the tumor markers (CA19-9 and CA125) of the patient.

A 66-year-old male received epigastric enhanced CT due to complaint of intermittent mid-upper abdominal pain. The CT showed 2.8×2.6 cm isodensity nodules in the tail of the pancreas. Enhanced CT scan showed that the enhancement degree was lower than normal pancreatic parenchyma (**Figure 1**, left). At the same time, multiple nodular low-density masses (**Figure 1C**, left) were detected in the liver. The tumor markers showed CA19-9 11.31 U/ml and CA125 74.73 U/ml. This patient received exploratory laparotomy. We found multiple metastases in the liver, peritoneum, and omentum during the operation. Rapid pathology revealed that the metastatic nodule was adenocarcinoma, and further immunohistochemistry showed pancreatic ductal adenocarcinoma (**Figure 2**). This patient received his first chemotherapy (GA regimen: gemcitabine 1,700 mg, nab-paclitaxel 200 mg) 20 days after operation; however, myelosuppression appeared after chemotherapy. This patient received subsequent two courses of chemotherapies after the physical condition was improved. At this time, whole abdominal enhanced CT showed the progression of liver metastasis (70 days after the operation) (**Figure 1B**, middle). The captured libraries from the abdominal metastasis of the patient were loaded onto a NovaSeq 6000 platform (Illumina) for 100 bp paired-end sequencing. The results showed that the patient had ATM germline mutation (gene type: heterozygote) and BRAF V600E mutation (mutation rate: 19.38%). In addition to the above two genes, no other common mutant genes were detected in this patient (**Table 1**). We decided to use the combined targeted therapy of vemurafenib and trametinib at the initial dose of vemurafenib 960 mg twice a day and trametinib 2 mg once a day. Initially, the therapy was tolerated well by the patient, but he began to develop rash and pruritus, which aggravated gradually after 3 months, so we stopped the treatment temporarily. After a week, his symptoms were relieved. At this time, the patient was given combined targeted therapy again, but the dose was adjusted to vemurafenib 720 mg twice a day and trametinib 2 mg once a day. As before, the patient showed tolerance at the beginning, and then the patient gradually developed rash and pruritus again, accompanied by oral ulcer and constipation. When the symptoms of the patient gradually worsened to intolerance, we stopped the drug again. This treatment cycle lasted 3 months, but at this time, the recovery time of the patient was significantly longer than before. Fourteen days later, we adjusted the dose of vemurafenib 480 mg twice a day and trametinib 2 mg once a day. This course lasted for 3 months, and the response of the patient was similar to that before. After this treatment, the patient recovered for 3 weeks and received combined targeted therapy for 3 months again. The dose was vemurafenib 240 mg twice a day and trametinib 2 mg once a day. Although the dose was gradually decreasing, the degree of complications was becoming more and more serious and the time for recovery was getting longer and longer. The patient had repeated fever and incomplete bowel obstruction due to constipation. After this treatment, the patient rested for 1 month, and then received vemurafenib 240 mg twice a day and trametinib 2 mg once a day again. However, this course lasted only 2 months, because the patient could no longer stand it. There was a decrease in glomerular filtration rate (low to 60 ml/min) and a continuous increase in urinary protein (up to 5.1 g/24 h). Finally, the patient rested for 2 months and received vemurafenib 240 mg twice a day and trametinib 2 mg once every 2 days for 1 month. At this time, we evaluated the tumor and found that the primary tumor was enlarged (**Figure 1**, right) and the liver had a new metastatic nodule (**Figure 1**), so the therapy was stopped. The patient was still in follow-up, and the tumor marker test results are shown in **Figure 3**. This patient received a total of 13 months of combined targeted therapy, and the treatment course lasted for 17 months. Excitingly, he had been followed for 24 months.

**Table 1:** Results of the genetic mutation test of the patient.

Chemotherapy-related test results		Target drug sites approved by the FDA		Immunotherapy-related test results	
Detection of gene (site)	Gene type	Detection of gene	Test result	Detection of gene	Test result
UGT1A1 (rs4148323)	GA	ALK	None	Immunohistochemical analysis of PD-L1	Negative (TPS = 0%, CPS = 0)
TPMT (rs1142345)	TT	BRAF	Pathogenic mutations	The mutation load	2.42 Muts/Mb (lower than 84% pancreatic cancer patients)
TPMT (rs1142345)	AA	BRCA1	None	Microsatellite analysis	Stable
DPYD (rs17376848)	AA	BRCA2	None	CD274 (PD-L1)	None
DPYD (rs1801159)	TT	CD274(PD-L1)	None	PDCD1LG2 (PD-L2)	None
CYP2C19 (rs4244285)	AA	EGFR	None	MLH1	None
		ERBB2	None	MSH2/6	None
		FGFR2	None	POLD1	None
		FGFR3	None	POLE	None
		KIT	None	BRIP1	None
		KRAS	None	TP53	None
		MET	None	ATM	Pathogenic mutations
		NRAS	None	ATR	None
		NTRK1	None	CHEK2	None
		NTRK2	None	FANCA	None
		NTRK3	None	RAD50	None
		PDGFRA	None	PALB2	None
		PIK3CA	None	CHEK1	None
		RET	None	MRE11A	None
		ROS1	None	PBRM1	None
				MDM2/4	None
				DNMT3A	None
				JAK1/2	None
				PTEN	None
				STK11	None
				CCND1	None
				FGFR19	None
				FGF3/4	None

TPS, positive percentage of tumor cells; CPS, composite positive score.

## Discussion

Over the past decade, the 5-year survival rate of PDAC has increased slightly from 5% to 8%, which almost results from the use of adjuvant and combination therapy rather than surgical techniques (13). However, due to the lack of effective targeted drugs, the precision medicine of PDAC obviously lags behind other malignant tumors (14). PDAC has four major gene mutations: *KRAS*, *TP53*, *CDKN2A*, and *Smad4* (14). The carcinogenic mutation of *KRAS* was found in more than 90% of PDAC (7, 15). This high prevalence rate has led to a considerable interest among scientists. Unfortunately, the KRAS protein has a high affinity for GTP and/or GDP, so it is difficult to be a target (16). For *TP53*, *CDKN2A*, and *Smad4*, there is also a lack of effective targeted therapy (17). In view of the lack of precision treatment for common mutations in PDAC, we believe that partial patients who have specific genetic mutations are the key for the precision treatment of PDAC. Although some PDAC patients, especially those with germline BRCA mutations, have received unprecedented benefits from Olaparib (18), the studies on PARP inhibitor mentioned so far have only focused on patients with germline or somatic BRCA1/2 or PALB2 mutations. This subgroup includes only about 14% of PDAC patients (19). Therefore, it is necessary for us to learn from the breakthrough findings of other cancers to benefit partial PDAC patients.

In 2002, whole-genome sequencing identified the *BRAF* gene as an oncogene and a potential therapeutic target for the first time (20). BRAF mutations occur in about 8% of human cancers, including melanoma, colorectal cancer, glioma, thyroid cancer, non-small cell lung cancer, cholangiocarcinoma, and several hematological malignancies (20, 21). Most mutations are caused by a missense mutation V600E in the kinase domain (22). BRAF V600E mutations increase the kinase activity of RAS and activate the MEK-ERK signal pathway (23). The first successful treatment for BRAF mutation was vemurafenib in melanoma (24, 25). Although the early and effective response to vemurafenib is common, drug resistance generally appears and largely limits the treatment time to 12 months (25). The drug resistance of BRAF inhibitors is largely due to the reactivation of MAPK signaling pathway (26), so combined targeted therapy is a reasonable approach. The specific method is to block the downstream signal of the RAS pathway by suppressing MEK1/2. Trametinib is the only MEK inhibitor approved as a monotherapy for metastatic melanoma (27). The combination of BRAF and MEK inhibitors has become the standard treatment for metastatic melanoma.

BRAF V600E mutation was found in about 3% of PDAC patients (8). At present, only a few data have reported the clinical experience of targeted therapy of BRAF mutation in advanced PDAC patients. Seghers et al. reported that a patient with PDAC in uncinate process of the pancreas with diffuse liver metastasis was treated with vemurafenib separately after failure of the first-line chemotherapy, resulting in a PFS of 9 months. Because the patient cannot tolerate the side effects of the MEK inhibitor cobimetinib, the PFS of the patient was shorter than that of the patient in this report (28). One case reported that a patient with stage IV pancreatic cancer with BRAF V600E mutation received combined targeted therapy with dabrafenib and trametinib, but the patient died of acute abdomen (29) after only 19 days of treatment, while the therapeutic effect of vemurafenib on advanced pancreatic cancer was not observed in another case (30). In addition, a patient with acinar cell carcinoma in the tail of the pancreas and BRAF V600E mutation obtained 8 months of PFS by combined target therapy with dabrafenib and trametinib, but the patient received chemotherapy for more than 5 years before the target therapy. Besides, the BRAF V600E mutation rate was only 1.4% (31), which was significantly lower than the 19.38% we reported here. In view of the fact that the patient has received combined targeted therapy and other treatments, it is difficult to attribute the benefit of the patient entirely to targeted therapy. A recent case also showed a positive response to combined targeted therapy in patients with pancreatic acinar cell carcinoma with BRAF V600E mutations, who achieved almost complete remission for 12 months (32). Considering that acinar cell carcinoma of the pancreas is a rare pancreatic exocrine tumor with more favorable prognosis than PDAC (33), the significance of combined targeted therapy for this tumor is less exciting than PDAC.

In this case, it is reported for the first time that the combination of BRAF inhibitors and MEK inhibitors can significantly benefit the survival of patients with stage IV PDAC, which makes us see the light of precision medicine in the treatment of PDAC. This case suggests that we should change the one-size-fits-all treatment concept of PDAC, and it is necessary to learn from the breakthrough findings of other cancer types. Compared with a previous similar report (28), our patient received a combination therapy, which is an important reason for prolonging the survival time (11). Current studies have not shown that intermittent treatment course is more beneficial to patients than continuous dosing (20). So, we think continuous dosing may be one of the reasons for the survival benefits of the patients. Based on this case, we found that the adverse reactions in the course of combination therapy were an important factor affecting the compliance of the patient. Although there were fewer adverse skin events in the full dose combination of the two drugs compared with BRAF inhibitor monotherapy, the addition of trametinib increased pyrexia, interstitial lung disease, venous thromboembolism, gastrointestinal bleeding, and heart (cardiomyopathy, decreased left ventricular ejection fraction) or ocular (retinal vein occlusion, uveitis) toxicity (34, 35). In this case, the patient showed repeated fever (remission after drug withdrawal), and after excluding the factors of infection, we believed that it is caused by the combination of drugs. In addition, patients with advanced melanoma who received combined targeted therapy showed a better response to PD-1 therapy (36), so triple therapy to combining targeted and immunotherapy may be a positive attempt.

## Data Availability Statement

The original contributions presented in the study are included in the article/supplementary material. Further inquiries can be directed to the corresponding author.

## Ethics Statement

Ethical review and approval were not required for the study on human participants in accordance with the local legislation and institutional requirements. The patients/participants provided their written informed consent to participate in this study.

## Author Contributions

NK and XL contributed to the study concept and design. ZW contributed to the investigation and writing of the original draft. DH contributed to the collection of pathology data and analysis. CC contributed to the collection of CT image data and analysis. All authors contributed to the article and approved the submitted version.

## Funding

This research was supported by Sichuan Province Science and Technology Planning Project (2020YFS0262), West China Hospital Clinical Research Incubation Project (21HXFH058), and the 1·3·5 Project for Disciplines of Excellence–Clinical Research Incubation Project (ZY2017302 and ZYJC21037), West China Hospital, Sichuan University.

## Conflict of Interest

The authors declare that the research was conducted in the absence of any commercial or financial relationships that could be construed as a potential conflict of interest.

## Publisher’s Note

All claims expressed in this article are solely those of the authors and do not necessarily represent those of their affiliated organizations, or those of the publisher, the editors and the reviewers. Any product that may be evaluated in this article, or claim that may be made by its manufacturer, is not guaranteed or endorsed by the publisher.
